# Clinical Applications and Mathematical Models of Bowel Sounds

**DOI:** 10.3390/biomedicines14030581

**Published:** 2026-03-05

**Authors:** Wanying Geng, Xinyuan Cao, Wanying Liao, Yingyun Yang

**Affiliations:** 1 4+4 Medical Doctor Program, Chinese Academy of Medical Sciences and Peking Union Medical College, Beijing 100730, China; gwy_0305@163.com; 2 Department of Gastroenterology, Department of Internal Medicine, Peking Union Medical College Hospital, Beijing 100730, China; caoxypumc@163.com (X.C.); liaowy17@mails.tsinghua.edu.cn (W.L.)

**Keywords:** bowel sounds, clinical applications, mathematical models

## Abstract

As a non-invasive, quantitative, and objective evaluation method, the analysis of bowel sounds has shown significant potential in clinical practice. In recent years, with the continuous advancement of signal processing techniques and analysis methods, research on bowel sounds has made significant progress. In this review, we discuss the main clinical applications of bowel sounds and summarize the commonly used analysis methods for bowel sounds at present. It is necessary to explore more comprehensive and effective signal processing technologies and methods in the future, and also establish a well-organized bowel sound database and scientific classification standards. This will promote better application of bowel sounds in clinical practice.

## 1. Introduction

Bowel sounds represent a type of physiological acoustic signal in humans, generated by the segmentation movements or peristalsis within the intestines. The friction and collision between intraluminal gases, liquids, and food residues with the intestinal walls, as well as interactions among these elements, produce intermittent gurgling or whooshing sounds. As indicators of gastrointestinal motility, bowel sounds have long been utilized in the diagnosis of intestinal diseases, forming part of standard clinical examinations. Cannon initiated the first scientific research on intestinal auscultation, revealing that the intestines produce rhythmic noises and continuous random noises with varying positions and intensities1. However, unlike heart or lung sounds, bowel sounds lack regularity and exhibit a stronger non-periodic and random nature, posing significant challenges for their detection and analysis.

Alterations in bowel sounds serve as crucial indicators of intestinal health. Under different pathological conditions, bowel sounds exhibit distinct characteristics. For instance, an increase in the frequency and volume of bowel sounds may suggest diseases like gastroenteritis or dysentery. If the frequency of bowel sounds is unusually high and loud enough to be heard without a stethoscope, it could indicate the early stages of diarrhea or intestinal obstruction. Conversely, a significant reduction or disappearance of bowel sounds, especially if no sounds are detected within 5 to 10 min, may be indicative of intestinal paralysis or acute peritonitis. Physicians can use these abnormal changes in bowel sounds to preliminarily assess a patient’s intestinal function, guiding subsequent diagnostic and therapeutic decisions.

Currently, endoscopic biopsy and imaging examinations are frequently used in the diagnosis of intestinal diseases, imposing a certain burden on the healthcare system and potentially causing pain, complications, infection, and radiation exposure risks to patients. In recent years, auscultation of bowel sounds has gained increasing attention from physicians due to its low cost, convenience, and safety. In-depth research into the characteristics of bowel sounds under various disease conditions and exploration of their relationship with intestinal diseases play an active role in disease diagnosis and prevention.

## 2. Literature Search Strategy

This review was conducted as a narrative synthesis of the existing literature on bowel sound analysis, with particular emphasis on clinical applications and mathematical modeling approaches.

A systematic search was performed in PubMed, Web of Science, and Scopus databases for articles published up to January 2025. The search terms included combinations of the following keywords: “bowel sounds”, “intestinal sounds”, “abdominal auscultation”, “phonoenterography”, “gastrointestinal motility”, “signal processing”, “spectral analysis”, “wavelet transform”, “adaptive filtering”, “machine learning”, and “artificial intelligence”. Reference lists of relevant articles were also manually screened to identify additional studies.

Studies were included if they:

(1) Investigated bowel sound acquisition, analysis, or modeling;
(2) Reported clinical applications related to gastrointestinal diseases or functional assessment; or
(3) Proposed mathematical or computational models for bowel sound signal processing.

We excluded:

(1) Conference abstracts without sufficient methodological description;
(2) Studies lacking primary data or methodological detail;
(3) Non-English publications.

Given the heterogeneity of study designs and the exploratory nature of many signal-processing investigations, a formal quantitative risk-of-bias assessment was not performed. However, methodological rigor, sample size, validation strategy (e.g., internal vs. external validation), and clinical relevance were qualitatively considered when synthesizing the evidence.

## 3. Clinical Application of Bowel Sounds

### 3.1. Diagnosis and Differentiation of Gastrointestinal Diseases

#### 3.1.1. Intestinal Obstruction

Bowel sound assessment has long been incorporated into the clinical evaluation of suspected intestinal obstruction; however, conventional auscultation is limited by subjectivity and poor reproducibility. Early objective evidence was provided by Yoshino et al., who applied computerized spectral analysis to patients with mechanical obstruction and identified three distinct frequency-based acoustic patterns that were not distinguishable by routine auscultation. Importantly, these patterns correlated with disease severity and clinical management, with higher-frequency profiles associated with earlier surgical intervention, suggesting that quantitative analysis may assist in stratifying obstruction severity and guiding treatment decisions [1]. Subsequent studies using electronic stethoscopes further explored the clinical relevance of acoustic parameters. Ching and Tan reported that although bowel sound analysis lacked specificity for diagnosing obstruction per se, temporal features such as sound duration and sound-to-sound intervals helped differentiate large from small bowel obstruction, with prolonged intervals associated with eventual surgical intervention [2]. In contrast, studies evaluating physician interpretation consistently demonstrated poor reproducibility of conventional auscultation. Durup-Dickenson et al. observed only slight-to-fair inter- and intra-observer agreement [3], and Breum et al. reported low sensitivity and modest specificity, concluding that bowel sounds alone should not guide clinical decision-making [4]. Collectively, these findings suggest that while traditional auscultation has limited standalone diagnostic value, quantitative and instrument-based bowel sound analysis may provide complementary information regarding obstruction severity and management strategies.

#### 3.1.2. Irritable Bowel Syndrome

Irritable Bowel Syndrome (IBS) is a prevalent functional gastrointestinal disorder, with symptoms including alterations in bowel movements, abdominal discomfort, and distension, which can markedly affect an individual’s quality of life [5,6]. It is estimated to impact roughly 11% of people worldwide [7].

Three studies from one research group [8,9,10] focused on the relationship between IBS and bowel sounds. Across a series of studies using electronic stethoscope-based recordings under fasting conditions, this group consistently demonstrated that IBS is not defined by distinctive sound morphology, but by altered temporal organization of bowel sound activity. In particular, fasting sound-to-sound intervals in IBS were markedly shorter than in healthy controls (approximately 500 ms vs. 1700 ms, *p* < 0.0001), achieving high diagnostic performance when interval-based thresholds were applied [8]. Importantly, this temporal abnormality appeared specific to functional disorders: patients with Crohn’s disease demonstrated longer intervals that overlapped with healthy controls rather than IBS [9]. An interval exceeding approximately 740 ms was associated with a high negative predictive value for IBS, supporting its role as an objective screening parameter [9]. Further methodological refinements incorporating spatial mapping confirmed reproducible regional patterns and modest spatial-frequency differences between functional bowel disorders and healthy subjects, enhancing physiological interpretability [10].

More recently, wearable sensors combined with machine learning have extended these findings into multivariate diagnostic frameworks. By integrating temporal and spectral features under fasting and postprandial conditions, contemporary models have achieved diagnostic performance approaching 90% sensitivity and specificity [11].

Overall, IBS-related abnormalities appear to be reflected primarily in bowel sound timing and organizational patterns rather than unique acoustic signatures, supporting bowel sound analysis as a promising noninvasive adjunct in IBS assessment.

#### 3.1.3. Inflammatory Bowel Disease

Inflammatory Bowel Disease (IBD) comprises a group of chronic systemic inflammatory diseases primarily affecting the gastrointestinal tract, including Crohn’s disease and ulcerative colitis [12].

Bowel sounds, as objective physiological signals reflecting intestinal motility and inflammatory status, hold potential clinical value in the diagnosis, disease monitoring, and long-term management of IBD. In diagnostic settings, multiple studies have demonstrated consistent and quantifiable differences in the temporal structure and spectral characteristics of bowel sounds between patients with IBD and healthy individuals. Baronetto et al. continuously collected abdominal acoustic signals from patients with IBD and healthy controls using wearable devices and applied automated analytical approaches, achieving high discriminative performance (AUC ≥ 0.83) across different digestive states and environmental noise conditions, suggesting that bowel sound analysis may serve as a noninvasive adjunct for screening and population stratification, particularly in resource-limited settings [13,14]. In differentiating IBD from IBS, Crohn’s disease is typically associated with prolonged bowel sound intervals, whereas IBS exhibits shorter and more regular intervals [8,15]. When combined with fecal calprotectin, acoustic features further improve diagnostic accuracy and may support outpatient triage [16]. Distinct acoustic characteristics have also been reported between IBD subtypes: Crohn’s disease may present with high-pitched “tinkling” sounds suggestive of strictures, whereas ulcerative colitis tends to show irregular and spasmodic patterns [17].

Regarding disease activity, active IBD is generally associated with hyperactive or dysrhythmic patterns, whereas remission states approximate normal acoustic profiles [18]. Analytical models based on bowel sound features have demonstrated high accuracy in identifying active disease (up to 96%) [19], and parameters such as entropy and spectral centroid correlate with endoscopic inflammation scores and fecal calprotectin levels [20]. In the context of complication monitoring, Crohn’s disease-related intestinal strictures may manifest as increases in the frequency and intensity of high-pitched bowel sounds, and continuous monitoring may facilitate early identification of partial bowel obstruction [3,21]. In ulcerative colitis, sustained attenuation or absence of bowel sounds may serve as an early warning sign of toxic megacolon. Postoperatively, the rate and pattern of bowel sound recovery reflect intestinal functional restoration in patients with IBD and may assist in monitoring postoperative complications and guiding dietary management [22]. During treatment and follow-up, effective therapy is associated with gradual transitions of bowel sound patterns from active to remission states, and longitudinal monitoring may help objectively evaluate treatment response and identify non-responders at an early stage [23]. Furthermore, subclinical alterations in bowel sounds preceding clinical relapse suggest a role for bowel sound monitoring in early flare prediction [14].

### 3.2. Monitoring Gastrointestinal Motility and Function

Monitoring bowel sounds can facilitate dynamic observation of gastrointestinal function, aiding in understanding the functional status of gastrointestinal motility, gas expulsion, and digestion [24,25].

As early as 1999, Tomomasa et al. demonstrated a close association between gastrointestinal sounds and the migrating motor complex (MMC), establishing a physiological basis for bowel sound analysis [26]. Subsequent studies by the same group showed that bowel sound analysis could reflect gastric emptying and intestinal peristalsis in pediatric populations; notably, infants with hypertrophic pyloric stenosis exhibited markedly reduced gastrointestinal sound activity prior to surgery (4.6 ± 1.0 mV/min) compared with healthy controls (31.7 ± 8.4 mV/min) [27].

Similarly, Kim et al. [28,29] conducted two studies in male patients with spinal cord injury and delayed gastric emptying. Using regression modeling and subsequently an artificial neural network approach based on acoustic features, they established and refined a colon transit time (CTT) prediction model, demonstrating the feasibility of quantitative acoustic estimation of gastrointestinal transit.

In the intensive care unit (ICU), bowel sounds may serve as a bedside indicator of gastrointestinal motility in critically ill patients. Earlier studies suggested that auscultation in the ICU requires improved standardization and equipment accuracy [30], and clinical judgment based solely on auscultation showed limited reliability [31]. Addressing these limitations, Sun et al. conducted a prospective observational study demonstrating the diagnostic value of continuous digital bowel sound monitoring for acute gastrointestinal injury (AGI). By integrating bowel sound parameters with clinical biomarkers, discriminant models were developed to differentiate AGI and stratify disease severity, with bowel sound rate identified as an independent risk factor [32].

### 3.3. Assessing Postoperative Recovery

General anesthesia can affect intestinal function, and the occurrence of postoperative ileus (POI) can lead to worsened prognosis, increased costs and prolonged hospital stays. A clinical study in 2014 showed that auscultation of bowel sounds was considered not useful for distinguishing between normal and pathological bowel sounds in patients [33]. However, recent studies have shown that bowel sounds can reflect postoperative intestinal recovery status as well as differences in gastrointestinal function before and after general anesthesia, providing guidance for postoperative feeding with high clinical value. Brennan M. R. Spiegel et al. used a disposable, non-invasive acoustic gastrointestinal surveillance (AGIS) biosensor to monitor and record bowel sounds. Initially, their study found that the average intestinal motility rates of healthy controls, the tolerated feeding group, and the POI group were 0.14, 0.03, and 0.016 contractions per second, respectively (*p* < 0.001). AGIS could differentiate post-abdominal surgery patients from healthy controls with 100% sensitivity and 97% specificity [34]. In a subsequent multicenter study, the results showed that AGIS predicted the onset of POI with sensitivity, specificity, and NPV of 63%, 72%, and 81%, respectively [35]. A recent study also demonstrated that general anesthesia weakened bowel sounds, which recovered to preoperative levels 3 h later [36]. Complementing these findings, Namikawa et al. evaluated a real-time bowel sound analysis system in patients undergoing gastric surgery and confirmed its feasibility for continuous quantitative perioperative assessment. Postoperative bowel sound recovery correlated inversely with operation time, supporting its role as an objective marker of peristalsis recovery [37]. Building on this concept, Shi et al. combined bowel sound–derived indices with clinical variables to develop a Bayesian model for predicting prolonged postoperative ileus (PPOI), demonstrating good discriminative ability and clinical net benefit [38]. In parallel, pharmacological modulation studies further emphasize the need for objective recovery markers. A meta-analysis showed that perioperative dexmedetomidine shortened time to first flatus and defecation after general anesthesia, although bowel sound–based endpoints were not directly assessed [39].

Overall, these findings support bowel sound monitoring as a complementary tool for evaluating surgical and pharmacological influences on postoperative gastrointestinal recovery.

### 3.4. Other Diseases

As early as 1983, a research team from Sweden enrolled 12 patients suspected of acute appendicitis undergoing appendectomy [40,41]. They believed that auscultation and recording of bowel sounds could help in the diagnosis of acute appendicitis. Similarly, Sugrue and Redfern [42] found that the average number of bowel sounds in normal subjects was higher than in appendicitis patients (*p* < 0.05), but there was no significant difference in the length of bowel sounds between the appendicitis group and the control group, indicating the diagnostic value of bowel sounds in acute appendicitis. Furthermore, Liatsos et al. [43] found that high-order cross (HOC) analysis of filtered and denoised bowel sounds can be used for the diagnosis of small-volume ascites. There was a significant difference between patients with cirrhosis accompanied by small-volume ascites and the control group (*p* < 0.0001), which was consistent with radiographic findings.

Beyond acute abdominal and intra-abdominal fluid-related conditions, alterations in bowel sounds have also been observed in systemic and neurodegenerative diseases associated with gastrointestinal dysmotility. A case–control study from Japan employing digital auscultation demonstrated that patients with Parkinson’s disease (PD) and multiple system atrophy (MSA) exhibited a significant reduction in both the frequency of bowel sounds and the cumulative duration of bowel sound activity per minute compared with healthy controls [44]. These findings suggest that bowel sound analysis may serve as a noninvasive marker of impaired gastrointestinal motor function secondary to autonomic and enteric nervous system involvement, thereby extending its potential clinical relevance beyond primary gastrointestinal pathology.

To clarify the classification of bowel sounds and their corresponding clinical value, the key characteristics, clinical implications and relevant literature of different types of bowel sounds are summarized in Table 1, and the summary of relevant clinical conditions and corresponding bowel sound characteristics is concluded in Table 2. The previous literature on the clinical application of bowel sounds is listed in Supplementary Table S1.

## 4. Establishment of a Mathematical Model for Bowel Sounds

In 1975, Dalle et al. pioneered the use of computational methods for the analysis of bowel sounds [47], conducting preliminary investigations into characteristics such as the frequency of bowel sounds, the effective total signal length, average energy, inter-sound intervals, and spectral distribution. With the rapid advancement of signal processing technology during the 1990s, research on bowel sounds garnered increased attention. The analysis of bowel sounds encompasses a multitude of parameters, including frequency, amplitude, duration, time intervals between successive bowel sounds, and the types of bowel sounds [46,48]. The primary challenges and focal points of research lie in the denoising of bowel sounds, feature extraction, and automatic classification. Commonly utilized analytical methodologies encompass spectral analysis, adaptive filtering, wavelet transform, principal component analysis, and artificial neural networks. (Table 3).

### 4.1. Spectral Analysis

Spectral analysis stands as a fundamental technique within the domain of signal processing, facilitating the transformation of time-domain signals into the frequency domain through Fourier analysis. Power spectral density estimation serves as a pivotal instrument in the spectral domain analysis, holding significant relevance in the processing of bowel sound signals. It translates the waveform of bowel sound signals, where amplitude varies with time, into a spectral plot where power varies with frequency.

During the 1990s, spectral analysis of bowel sounds through Fast Fourier Transform (FFT) and Autoregressive (AR) models categorized them into four distinct types: normal sounds, gurgles, high-pitched sounds, and metallic sounds. Gurgles, indicative of the passage of gas through fluid, have a longer duration of about 100 milliseconds and a lower frequency range, typically between 60 and 200 Hz. High-pitched sounds have a similar duration to normal sounds, with potential multiple peaks in the frequency domain, the most prominent being between 300 and 600 Hz. Metallic sounds last about 50 milliseconds and are characterized by three or more peaks, with the primary peak generally ranging from 600 to 1000 Hz, and other peaks distributed between 300 and 1000 Hz. Bray et al., through spectral analysis, also reached analogous conclusions, indicating that the frequency range for normal sounds is 100 to 1000 Hz, with a duration of 5 to 200 milliseconds; the typical high-pitched sounds have a frequency of 500 to 700 Hz, with a duration of 5 to 20 milliseconds [45]. Beyond static spectral characterization, Haraguchi and Emoto introduced a stimulus–response analytical framework based on bowel sounds to quantify gastrointestinal motor reactions to physiological drinking stimuli. By relating time-domain features of bowel sounds before ingestion to post-ingestion changes, their approach captured dynamic interactions between baseline motility and stimulus-induced responses, extending spectral analysis toward functional assessment of gastrointestinal reactivity [49].

### 4.2. Adaptive Filtering

Adaptive filtering is distinguished by two principal features: first, its capacity for automatic parameter adjustment through a “learning” process, and second, its ability to “track” changes in the statistical properties of the input signal and adjust parameters accordingly. This technique operates effectively in the absence of prior knowledge, utilizing real-time collected data and adhering to the principle of minimal error to automatically fine-tune weight factors, thereby minimizing the discrepancy between the source signal and the outcome. Adaptive filtering is distinguished by two principal features: first, its capacity for automatic parameter adjustment through a “learning” process, and second, its ability to “track” changes in the statistical properties of the input signal and adjust parameters accordingly. Mansy et al. employed adaptive filters to enhance bowel sound signals, with experimental data sourced from rats. Their research indicated that adaptive filtering effectively removed noise from bowel sound signals, thereby enhancing the audibility of the sounds, and this method holds instructive significance for noise reduction in human applications [46]. Building upon classical adaptive filtering, Wang et al. developed a flexible dual-channel digital auscultation patch incorporating active noise reduction for long-term bowel sound monitoring in noisy clinical environments. By integrating adaptive filtering with multichannel cross-validation, the system effectively suppressed both ambient and nonstationary noise, enabling reliable continuous acquisition of bowel sounds and addressing key technical barriers to wearable bowel sound monitoring in real-world settings [50].

### 4.3. Wavelet Transform

The wavelet transform serves as a mathematical microscope with capabilities for magnification, reduction, and translation, akin to a set of bandpass filters with fixed bandwidth and variable center frequencies [51]. Wavelet analysis involves the selection of an appropriate mother wavelet and generates “wavelets” through binary dilations and translations. Employing short windows at high frequencies and long windows at low frequencies reflects the philosophy of relative bandwidth frequency analysis and adaptive variable resolution analysis. Leontios [52] utilized a wavelet-based filter to enhance bowel sounds, constructing a Stationary-Nonstationary Wavelet Transform filter (WTST-NST) by combining multiresolution analysis and hard thresholding, effectively separating and enhancing bowel sounds from noise. Dimoulas et al. proposed a wavelet-domain Wiener filtering method for denoising [53], which integrates the discrete wavelet transform, wavelet packets, and Wiener filtering for the analysis and processing of bowel sounds, a method that is also applicable to other human sound signals.

### 4.4. Principal Component Analysis (PCA)

Principal Component Analysis (PCA) is an exploratory statistical technique that consolidates dispersed information across a set of variables into a few composite indicators, known as principal components. Ranta et al. introduced a bowel sound analysis approach grounded in PCA, which, coupled with wavelet denoising, segmentation, and spatial localization [54], aids in reducing spatial dimensions and eliminating redundancy. However, as an emerging analytical method, the application of PCA has several limitations that require further exploration and refinement.

### 4.5. Machine Learning-Based Models

With the maturation of bowel sound signal preprocessing and feature extraction techniques, machine learning has emerged as a central methodological paradigm for constructing mathematical models of bowel sounds. Compared with conventional rule-based or single-parameter analyses, machine learning based models enable the integration of multidimensional acoustic features and the characterization of complex, nonlinear relationships underlying gastrointestinal motility. Importantly, these approaches have shifted bowel sound analysis from descriptive acoustic characterization toward predictive and clinically actionable modeling frameworks.

Early machine learning applications in bowel sound analysis primarily focused on supervised classification using handcrafted features. Dimoulas et al. combined wavelet-based feature extraction with artificial neural networks to develop the Automated Intestinal Motility Analysis System (AIMAS), demonstrating robust adaptability across different model parameters and favorable classification performance in distinguishing bowel sound patterns [55]. In parallel, probabilistic classifiers such as Naive Bayes models were employed to categorize bowel sound types based on acoustic feature distributions, providing a computationally efficient framework for automated bowel sound classification [57]. These studies established artificial neural networks and probabilistic learning models as foundational tools for bowel sound modeling and laid the groundwork for more advanced AI-driven systems.

Beyond model selection, machine learning-based bowel sound analysis has increasingly emphasized structured mathematical representations of intestinal activity. A widely adopted modeling framework describes bowel sounds using four quantitative intestinal activity parameters: Individual Wave Components (IWC), Pressure Index (PI), Component Quantity (CQ), and Component Interval Time (CIT). Based on these parameters, bowel sounds are categorized into Single Bursts (SB), Multiple Bursts (MB), Continuous Random Sounds (CRS), and Harmonic Sounds (HS), providing a standardized mathematical description that links acoustic patterns with underlying physiological processes [57]. This parameterized representation enhances model interpretability and facilitates cross-study comparability, addressing one of the major barriers to clinical translation.

More recent methodological advances have shifted toward integrated machine learning pipelines that incorporate feature engineering, temporal modeling, and ensemble learning strategies. Burne et al. proposed an ensemble-based automated bowel sound detection framework that combined handcrafted acoustic features with one- and two-dimensional representations derived from mel-frequency cepstral coefficients. Importantly, a hierarchical hidden semi-Markov model was introduced to explicitly model temporal dependencies and event segmentation, highlighting the methodological advantage of sequence-aware modeling over static classifiers [58]. Such temporal architectures are particularly relevant given the inherently episodic and nonstationary nature of bowel sounds.

Machine learning models have also been extended from detection and classification toward clinically oriented prediction tasks. Liu et al. developed a machine learning–assisted bowel sound analysis framework to predict early enteral nutrition–associated diarrhea in patients with acute pancreatitis. Their approach integrated feature selection, regression-based identification of key predictors, ensemble modeling, and partial dependence analysis to enhance model interpretability, exemplifying a structured methodological pathway from acoustic signal analysis to clinically meaningful risk modeling [56]. These developments illustrate how AI-based bowel sound analysis is evolving into a tool for risk stratification and decision support rather than merely signal categorization.

From a translational perspective, AI-driven bowel sound analysis holds significant potential for real-world clinical implementation. When combined with wearable or patch-based acoustic sensors, embedded machine learning algorithms may enable continuous, bedside, or even remote monitoring of gastrointestinal motility in postoperative wards, intensive care units, and outpatient settings. Such systems could support early detection of postoperative ileus, acute gastrointestinal injury, inflammatory bowel disease flares, and enteral nutrition intolerance, thereby facilitating timely intervention and individualized management strategies. Integration with electronic health records and multimodal clinical biomarkers may further enhance predictive performance and enable precision-medicine–oriented applications.

However, several challenges remain before widespread clinical adoption can be achieved. Many current models are developed using relatively small, single-center datasets, limiting external validity and generalizability. Prospective multicenter validation with standardized acquisition protocols is essential. In addition, variability in sensor placement, environmental noise, and hardware platforms may substantially influence acoustic features, underscoring the need for standardized recording frameworks and device calibration. Model interpretability also remains critical; clinicians may be reluctant to rely on opaque “black-box” algorithms without clear physiological correlations. The incorporation of explainable AI techniques and physiologically informed modeling strategies may improve transparency and clinical acceptance. Furthermore, regulatory approval, data governance, and reproducibility standards must be addressed before AI-based bowel sound systems can be integrated into routine diagnostic or prognostic workflows.

Collectively, machine learning-based mathematical models for bowel sounds are characterized by their ability to integrate multidomain acoustic features, capture nonlinear and temporal dynamics, and support standardized quantitative descriptions of gastrointestinal motility. As these models evolve from classification tools toward validated clinical decision-support systems, large-scale prospective studies, external validation, and standardized reporting guidelines will be essential to fully realize their translational potential.

## 5. Discussion

Bowel sounds reflect the dynamic interplay between gastrointestinal motility, intraluminal contents, and neuromuscular regulation, yet their clinical value has long been underestimated because of their irregular and nonstationary nature. The studies reviewed here demonstrate that, when analyzed quantitatively, bowel sounds convey reproducible and clinically meaningful information across a wide range of gastrointestinal conditions. This body of evidence supports a gradual shift from subjective auscultation toward objective and model-based interpretation.

Across different disease settings, consistent alterations in bowel sound characteristics have been reported. Changes in sound frequency, temporal distribution, and spectral composition distinguish patients with intestinal obstruction, irritable bowel syndrome, inflammatory bowel disease, postoperative ileus, and gastrointestinal dysfunction in critically ill patients from healthy individuals. Many of these differences are subtle and difficult to recognize through conventional auscultation, which remains limited by poor inter-observer agreement and susceptibility to environmental noise. These limitations have motivated the development of instrument-based acquisition and quantitative analytical methods as a more reliable means of assessing bowel sounds.

Methodological advances have played a central role in enabling this transition. Early studies focused on time-domain and frequency-domain descriptions of bowel sounds, providing initial insight into their acoustic structure. As research moved into more complex clinical environments, the need for robust signal extraction became increasingly apparent. The introduction of adaptive filtering and wavelet-based denoising substantially improved signal quality and event detection, allowing bowel sound analysis to extend beyond controlled laboratory settings. Building on these foundations, machine learning approaches enabled the integration of multidimensional acoustic features and more accurate modeling of the nonlinear and dynamic characteristics of gastrointestinal activity. Parameterized and temporal modeling frameworks further linked acoustic patterns to physiologically meaningful indices and accounted for the episodic nature of bowel sounds, thereby extending bowel sound analysis from basic signal classification toward clinically oriented prediction and functional assessment.

Despite these advances, several barriers remain before bowel sound analysis can be routinely incorporated into clinical practice. Substantial heterogeneity in recording protocols, sensor placement, acquisition duration, and preprocessing strategies limits comparability across studies and hampers external validation. In addition, many models are developed using relatively small and single-center datasets, raising concerns regarding robustness and generalizability. From a clinical standpoint, further work is also needed to improve the interpretability of machine learning models and to clarify how bowel sound-based metrics can be integrated into existing diagnostic pathways.

Looking ahead, future research should focus on standardizing acquisition and analytical frameworks, conducting prospective multicenter validation, and integrating bowel sound features with clinical variables and biomarkers. Bowel sound analysis is best viewed as a complementary, noninvasive approach that enables continuous and repeatable assessment of gastrointestinal function. With continued methodological refinement and clinical validation, quantitative bowel sound analysis may play an increasing role in the longitudinal management of gastrointestinal disorders.

## 6. Physiological and Technical Confounders in Bowel Sound Analysis

Bowel sound signals are inherently context-dependent physiological phenomena and may be influenced by a range of physiological and technical variables that require careful consideration in both experimental design and model development. Acoustic characteristics vary according to sensor placement, as different abdominal regions correspond to distinct intestinal segments with heterogeneous motility patterns. Variability introduced by recording location can affect amplitude, frequency distribution, and temporal dynamics. Nevertheless, prior studies have demonstrated that predefined anatomical recording sites, standardized multi-point acquisition protocols, and more recently wearable multi-sensor arrays can substantially improve reproducibility and mitigate location-related bias.

Interindividual differences in body habitus, particularly BMI and abdominal wall fat thickness, also influence acoustic transmission by attenuating signal intensity and altering spectral properties. While such factors may reduce signal-to-noise ratio, advances in amplification techniques, adaptive filtering, and feature normalization have shown that meaningful signals remain detectable across diverse patient populations. Importantly, anthropometric parameters can be incorporated as covariates in machine learning frameworks, allowing models to adjust for systematic interindividual variability rather than treating it as unstructured noise.

Clinical context further modulates bowel sound generation. Postoperative scars, altered anatomy, and perioperative inflammatory states may modify both sound propagation and intrinsic motility patterns. However, several investigations conducted specifically in postoperative settings have confirmed the feasibility of bowel sound monitoring for assessing recovery of gastrointestinal function, suggesting that although variability increases, clinically relevant acoustic information persists under standardized recording conditions. Similarly, physiological state plays a central role: fasting and postprandial motility patterns differ substantially due to the migrating motor complex and fed-pattern peristalsis. Many existing studies have therefore adopted controlled fasting durations or standardized meal challenges, indicating that nutritional state–related variability can be effectively minimized through protocol design.

Additional factors such as bowel gas volume, stool characteristics, and medication exposure—including opioids, anticholinergics, and anesthetic agents—also influence acoustic signatures by altering motility and intraluminal dynamics. While these elements introduce complexity, they are not inherently prohibitive. When systematically documented and integrated as structured metadata, they may instead enhance model interpretability by linking acoustic changes to physiological or pharmacological mechanisms. In longitudinal monitoring scenarios, within-subject comparisons further reduce the impact of baseline variability.

Collectively, these considerations highlight that bowel sound variability arises from identifiable and, to a considerable extent, controllable sources. Rather than representing insurmountable barriers to clinical implementation, these confounders underscore the importance of standardized acquisition protocols, comprehensive metadata collection, and modeling strategies that explicitly account for physiological context. Future multicenter studies should therefore combine rigorous protocol harmonization with robust machine learning techniques to improve generalizability and facilitate translation from experimental research to real-world clinical practice.

## 7. Novel Contribution of This Review

Although previous reviews have discussed bowel sound analysis from either a clinical or engineering perspective, most have focused predominantly on signal processing techniques, wearable device development, or specific disease applications in isolation. Few studies have systematically integrated clinical applications and mathematical modeling frameworks into a unified translational perspective.

This Review provides several novel contributions. First, we propose a clinically oriented framework that links bowel sound characteristics across different gastrointestinal diseases with corresponding analytical methodologies, thereby bridging bedside clinical interpretation and computational modeling. Rather than presenting clinical findings and mathematical models in parallel, we synthesize how specific acoustic features (e.g., temporal intervals, spectral distribution, entropy, and activity indices) are operationalized within modeling pipelines and translated into clinically meaningful endpoints such as disease stratification, activity assessment, and postoperative recovery prediction.

Second, we provide a structured categorization of modeling strategies—from classical spectral and wavelet-based methods to contemporary machine learning and temporal-sequence modeling—highlighting their methodological evolution and clinical applicability. By organizing these approaches within a comparative framework (Table 2), we clarify how different analytical paradigms address specific physiological and diagnostic questions.

Third, this Review emphasizes the emerging shift from static classification toward dynamic, longitudinal, and predictive modeling of gastrointestinal function. We discuss how bowel sound analysis is transitioning from simple pattern recognition to risk stratification, complication prediction, and integration with multimodal biomarkers, thereby outlining a forward-looking translational roadmap.

Finally, we identify key barriers to clinical implementation, including heterogeneity in acquisition protocols, limited multicenter validation, and a lack of standardized classification systems. By articulating these gaps within a structured clinical–computational continuum, this Review provides additional conceptual value beyond previous narrative summaries and aims to guide future methodological standardization and translational research.

## Figures and Tables

**Table 1:** Summary of Different Bowel Sounds, Their Characteristics and Clinical Meanings.

Type of Bowel Sound	Specific Characteristics (Frequency, Intensity, Pitch, Rhythm)	Clinical Meanings	Relevant Literature
Normal Bowel Sounds	Frequency: 100–1000 Hz; Duration: 5–200 ms; Intensity: Moderate; Rhythm: Intermittent, regular; No obvious high-pitched or metallic components	Indicates normal gastrointestinal motility and intestinal function, no obvious organic or functional abnormalities	[45]
Gurgles	Frequency: 60–200 Hz; Duration: ~100 ms; Intensity: Mild to moderate; Rhythm: Intermittent; Associated with gas passage through intestinal fluid	Common in normal individuals after meals; excessive gurgles may suggest increased intestinal peristalsis (e.g., early gastroenteritis)	[46]
High-pitched Bowel Sounds	Frequency: 300–700 Hz; Duration: 5–20 ms (similar to normal sounds); Intensity: High; Rhythm: May be frequent; Multiple frequency peaks possible	Suggests increased intestinal peristalsis or mild obstruction; common in IBS, early intestinal obstruction, or gastroenteritis	[45,46]
Metallic Bowel Sounds	Frequency: 600–1000 Hz (primary peak); Duration: ~50 ms; Intensity: High; Rhythm: Intermittent; ≥3 frequency peaks	Highly suggestive of intestinal obstruction (especially mechanical obstruction); may also occur in intestinal strictures (e.g., Crohn’s disease)	[46]
Diminished/Absent Bowel Sounds	Frequency: <5 sounds per minute; Intensity: Weak or inaudible; Rhythm: Irregular or absent; No obvious acoustic features	Indicates decreased intestinal motility; common in intestinal paralysis, acute peritonitis, postoperative ileus, or advanced IBD	[32,44]

**Table 2:** Summary of Relevant Clinical Conditions and Corresponding Bowel Sound Characteristics.

Clinical Condition	Summary of Bowel Sound Characteristics	Key Clinical Implications	Relevant Literature
**Intestinal Obstruction (Mechanical)**	Metallic/high-pitched sounds; 3 frequency-based patterns; higher frequency associated with severe obstruction; sound duration/dominant frequency differ between small/large bowel obstruction	Quantitative analysis helps stratify severity and guide surgical decisions; subjective auscultation has poor reproducibility	[1,3,4]
**Irritable Bowel Syndrome (IBS)**	Shorter fasting sound-to-sound intervals (~500 ms vs. 1700 ms in controls); no distinctive morphology; altered temporal organization; improved diagnostic accuracy with multi-feature models (sensitivity/specificity ~90%)	Bowel sound timing serves as an objective parameter for diagnosis and differentiation from organic diseases	[8,9,11]
**Inflammatory Bowel Disease (IBD)**	Crohn’s disease: Prolonged sound intervals (overlapping with controls), high-pitched/metallic sounds (strictures); Ulcerative colitis: Spasmodic/irregular sounds; Active IBD: Hyperactive/dysrhythmic sounds; Remission: Near-normal sounds	Aids differentiation from IBS; an objective marker of disease activity and stricture monitoring	[9,14,17]
**Postoperative Ileus (POI)**	Diminished/absent sounds in early stage; recovery correlates with increased sound counts; motility rate lower than normal recovery group (0.016 vs. 0.03 contractions/sec)	Objective marker of intestinal recovery; helps predict POI and guide postoperative feeding	[34,35,38]
**Critically Ill Patients (AGI)**	Dysrhythmic sounds; sound rate independently predicts AGI and severity; poor interpretation accuracy by clinicians	Continuous digital monitoring provides an objective bedside assessment of gastrointestinal dysfunction	[31,32]
**Parkinson’s Disease (PD)**/MSA	Reduced frequency and cumulative duration of sounds; diminished sound activity; slower rhythm	Noninvasive marker of gastrointestinal dysmotility secondary to autonomic nervous system involvement	[44]

**Table 3:** Summary of the Mathematical Model in bowel sounds analysis and its Clinical Relevance.

Methodological Category	Core Techniques	Key Modeling Elements	Methodological Contribution	Relevance to Clinical Conditions	Representative References
**Spectral Analysis**	FFT, AR models, power spectral density estimation	Dominant frequency bands, spectral peaks, signal duration	Frequency-domain characterization of bowel sound types and basic signal classification	Classifies obstruction-related sound patterns; differentiates normal vs. abnormal sounds; assesses obstruction severity	[1,45,49,50]
**Adaptive Filtering**	LMS-based adaptive filters, dual-channel noise cancellation	Real-time parameter adaptation, nonstationary noise suppression	Enhancement of bowel sound signals and robust denoising in noisy environments	Enhances sound signals in noisy clinical settings; improves signal quality for subsequent analysis	[50,51]
**Wavelet Transform**	Discrete wavelet transform, wavelet packets, Wiener filtering	Time–frequency localization, multiresolution decomposition	Effective separation of bowel sounds from noise and extraction of transient acoustic features	Denoises bowel sounds; extracts features for IBD/POI diagnosis; aids in small-volume ascites detection	[43,52,53,54]
**Principal Component Analysis (PCA)**	Dimensionality reduction, feature decorrelation	Principal components of multichannel acoustic features	Reduction in feature redundancy and facilitation of spatial and temporal modeling	Estimates colon transit time; diagnoses IBS/IBD; predicts early enteral nutrition-associated diarrhea	[29,55,56]
**Machine learning-based Models**	Artificial neural networks, Naive Bayes classifiers, ensemble learning, hidden semi-Markov models	Handcrafted acoustic features, MFCC-based representations, temporal dependency modeling	Integration of multidimensional features, nonlinear classification, and sequence-aware bowel sound modeling	Detects neonatal bowel sounds; predicts POI; improves diagnostic accuracy of functional GI disorders	[38,56,57,58]

## Data Availability

No new data were created or analyzed in this study.

## Supplementary Materials

The following supporting information can be downloaded at: https://www.mdpi.com/article/10.3390/biomedicines14030581/s1, Table S1: Summary of representative studies on the clinical application of bowel sounds.
