# Supplementary material for: Clinical Applications and Mathematical Models of Bowel Sounds
Source: Biomedicines. 2026 Mar 5;14(3):581. doi: 10.3390/biomedicines14030581 (PMC13023620; doi:10.3390/biomedicines14030581)
Supplement: Supplementary file 1 [file biomedicines-14-00581-s001.zip › biomedicines-4158760-supplementary.pdf]

Supplementary Table S1. Summary of representative studies on the clinical application of bowel sounds

Study (Year)	Target condition / population	Methodology	Main findings	Clinical implication
**Yoshino et al. (1990) [1]**	Mechanical intestinal obstruction	Computerized spectral analysis of bowel sounds	Bowel sounds were classified into three frequency-based types; higher-frequency patterns were associated with increased surgical intervention and shorter time to operation	Quantitative bowel sound analysis may reflect obstruction severity and assist treatment stratification
**Ching & Tan (2012) [3]**	Acute and subacute small and large bowel obstruction	Electronic stethoscope with spectral feature analysis	Overall diagnostic specificity was limited; sound duration and dominant frequency differed between small and large bowel obstruction; prolonged sound intervals predicted need for surgery in SBO	Bowel sound features may help localize obstruction and identify patients requiring surgery
**Durup-Dickenson et al. (2013) [2]**	Healthy volunteers and emergency patients	Blinded physician interpretation of recorded bowel sounds	Inter- and intra-observer agreement for pitch, intensity, and quantity was only slight to fair (κ = 0.19–0.30)	Highlights poor reproducibility of subjective auscultation
**Breum et al. (2015) [4]**	Suspected bowel obstruction	Recorded bowel sounds interpreted by junior and senior doctors	Low sensitivity (median 0.42), modest specificity, and poor inter-observer agreement; no difference by experience level	Auscultation alone should not guide clinical decisions in suspected obstruction
**Craine et al. (1999) [8]**	Irritable bowel syndrome (IBS)	Digitized bowel sound interval analysis	IBS patients showed significantly shorter sound-to-sound intervals during fasting	Objective bowel sound metrics may aid IBS diagnosis
**Craine et al. (2001) [9]**	IBS vs Crohn's disease	Enterotachogram-based sound interval analysis	Longer sound intervals in Crohn's disease than IBS; intervals >740 ms effectively excluded IBS	Supports bowel sound intervals in differential diagnosis
**Craine et al. (2002) [10]**	IBS and non-ulcer dyspepsia	Two-dimensional bowel sound mapping	Distinct spatial and temporal sound patterns among functional GI disorders	Bowel sound mapping may assist phenotyping of functional GI diseases
**Du et al. (2019) [11]**	IBS	Automated bowel sound feature model	Sensitivity and specificity >85% for IBS diagnosis	Proof of concept for noninvasive, quantitative IBS assessment

Study (Year)	Target condition / population	Methodology	Main findings	Clinical implication
**Tomomasa et al. (1999) [26]**	Healthy fasted adults	Simultaneous bowel sound recording and manometry	Gastrointestinal sounds correlated with migrating motor complex (MMC) activity	Established physiological basis of bowel sound analysis
**Tomomasa et al. (1999) [27]**	Infants with hypertrophic pyloric stenosis	Quantitative bowel sound energy analysis	Markedly reduced bowel sounds before surgery, with recovery after pyloromyotomy	Reflects gastric emptying and peristalsis in pediatric disease
**Kim et al. (2011) [29]**	Patients with delayed gastric emptying	Regression and neural network models based on sound features	Bowel sounds enabled noninvasive estimation of colon transit time	Potential alternative to radiologic motility assessment
**Li et al. (2012) [30]**	Critically ill ICU patients	Clinical bowel sound monitoring	Bowel sounds reflected GI motility but lacked objectivity	Emphasized need for standardized digital monitoring
**Li et al. (2014) [31]**	Comatose ICU patients	Survey and clinical observation	Low accuracy of bowel sound interpretation by nurses and physicians	Reinforced limitations of conventional auscultation
**Sun et al. (2024) [32]**	Critically ill patients with AGI	Continuous digital bowel sound monitoring	Bowel sound rate independently predicted AGI and disease severity	Objective bedside tool for GI dysfunction assessment in ICU
**Spiegel et al. (2014) [34]**	Postoperative ileus (POI)	Acoustic gastrointestinal surveillance (AGIS) biosensor	Clear differentiation among healthy controls, normal recovery, and POI	Enables objective monitoring of postoperative GI recovery
**Kaneshiro et al. (2016) [35]**	Postoperative GI recovery	Multicenter AGIS validation	High negative predictive value (81%) for ruling out POI	Supports early feeding decisions
**Namikawa et al. (2021) [37]**	Patients undergoing gastric surgery	Real-time bowel sound monitoring	Postoperative bowel sound recovery correlated with operative factors	Objective marker of postoperative peristalsis recovery
**Shi et al. (2024) [38]**	Gastric cancer patients after gastrectomy	Integrated bowel sound indices and clinical variables	Accurate prediction of prolonged postoperative ileus	Supports bowel sound–based risk stratification
**Arnbjörnsson et al. (1983) [40]**	Suspected acute appendicitis	Auscultation and sound recording	Altered bowel sounds observed in appendicitis	Early exploration of diagnostic utility

Study (Year)	Target condition / population	Methodology	Main findings	Clinical implication
**Sugrue & Redfern (1994) [42]**	Acute appendicitis	Computerized phonoenterography	Reduced bowel sound frequency in appendicitis patients	Suggests supportive diagnostic value
**Liatsos et al. (2003) [43]**	Cirrhosis with small-volume ascites	High-order cross analysis of bowel sounds	Significant differences compared with controls	Noninvasive detection of occult ascites
**Ozawa et al. (2011) [44]**	Parkinson's disease and MSA	Quantitative bowel sound analysis	Reduced bowel sound number and duration	
